# Identification of an immune‐related gene signature as a prognostic target and the immune microenvironment for adrenocortical carcinoma

**DOI:** 10.1002/iid3.680

**Published:** 2022-08-17

**Authors:** Chengdang Xu, Caipeng Qin, Jingang Jian, Yun Peng, Xinan Wang, Xi Chen, Denglong Wu, Yuxuan Song

**Affiliations:** ^1^ Department of Urology, Tongji Hospital, School of Medicine Tongji University Shanghai China; ^2^ Department of Urology Peking University People's Hospital Beijing China; ^3^ Department of Urology, The First Affiliated Hospital of Soochow University, Dushu Lake Hospital Affiliated to Soochow University Suzhou Medical College of Soochow University Suzhou China; ^4^ Department of Urology Tianjin Medical University General Hospital Tianjin China

**Keywords:** adrenocortical carcinoma, immunology, prognosis, signatures, tumor microenvironment

## Abstract

**Background:**

Adrenocortical carcinoma (ACC) is a rare endocrine malignancy. Even with complete tumor resection and adjuvant therapies, the prognosis of patients with ACC remains unsatisfactory. In the microtumor environment, the impact of a disordered immune system and abnormal immune responses is enormous. To improve treatment, novel prognostic predictors and treatment targets for ACC need to be identified. Hence, credible prognostic biomarkers of immune‐associated genes (IRGs) should be explored and developed.

**Material and methods:**

We downloaded RNA‐sequencing data and clinical data from The Cancer Genome Atlas (TCGA) data set, Genotype‐Tissue Expression data set, and Gene Expression Omnibus data set. Gene set enrichment analysis (GSEA) was applied to reveal the potential functions of differentially expressed genes.

**Results:**

GSEA indicated an association between ACC and immune‐related functions. We obtained 332 IRGs and constructed a prognostic signature on the strength of 3 IRGs (*INHBA*, *HELLS*, and *HDAC4*) in the training cohort. The high‐risk group had significantly poorer overall survival than the low‐risk group (*p* < .001). Multivariate Cox regression was performed with the signature as an independent prognostic indicator for ACC. The testing cohort and the entire TCGA ACC cohort were utilized to validate these findings. Moreover, external validation was conducted in the GSE10927 and GSE19750 cohorts. The tumor‐infiltrating immune cells analysis indicated that the quantity of T cells, natural killer cells, macrophage cells, myeloid dendritic cells, and mast cells in the immune microenvironment differed between the low‐risk and high‐risk groups.

**Conclusion:**

Our three‐IRG prognostic signature and the three IRGs can be used as prognostic indicators and potential immunotherapeutic targets for ACC. Inhibitors of the three novel IRGs might activate immune cells and play a synergistic role in combination therapy with immunotherapy for ACC in the future.

## Graphical abstract

In the present study, we aimed to identify an immune‐related gene (IRG) prognostic signature and find potential immunotherapeutic targets for adrenocortical carcinoma (ACC) patients. In addition, what we found is that three‐IRG prognostic signature and the three IRGs could serve as prognostic indicators and potential immunotherapeutic targets for ACC.

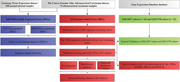

## 1 INTRODUCTION

Adrenocortical carcinoma (ACC) is a rare endocrine malignancy worldwide.^1^, ^2^ The incidence is reported to be one to two per million per year. The prognosis is poor with a median overall survival (OS) of 3–4 years.^3^ Even with complete tumor resection and adjuvant therapies, the prognosis of patients with ACC following treatment remains unsatisfactory.^4^ The risk of recurrence and metastasis ranged from at least 15% to 40% among patients with ACC after initial treatment.^5^, ^6^ Consequently, novel prognostic predictors and treatment targets for ACC are needed and credible prognostic biomarkers of immune‐associated genes (IRGs) should be explored.

In the microtumor environment, the impact of a disordered immune system and abnormal immune responses is enormous.^7^ Immune cells may modify the signaling molecule to influence the immunological response, which significantly impacts the risk of recurrence, change, and diffusion of the neoplasm.^8^, ^9^ Previous research studies have reported that some tumor cells may evade immune surveillance and immune elimination, resulting in tumor infiltration and metastasis.^10^ An abnormal immune state is thought to be associated with glioblastoma progression.^11^ Martinez‐Bosch et al.^10^ suggested that immunosuppressive macrophages and myeloid‐derived suppressive cells may significantly suppress the immune microenvironment in pancreatic cancer. Peng et al.^12^ identified two immune‐related genes could serve as potential biomarkers for immunotherapy in ACC, which provide better insights into ACC microenvironment. In addition, N6‐methyladenosine methylation regulators had impacts on ACC prognosis through regulating immune‐related functions.^13^ Bioinformatics analyses based on The Cancer Genome Atlas (TCGA) constructed a competitive endogenous RNA network associated with tumor‐infiltrating immune cells and determined that the immune system is implicated in the microenvironment of ACC.^14^

Aberrantly expressed IRGs may be potential therapeutic targets and prognostic biomarkers for patients with ACC. For this purpose, we explored IRGs and constructed an IRG prognostic signature. This study was designed using the gene expression profiles from the TCGA ACC and Genotype‐Tissue Expression (GTEx) data sets. To investigate the IRG signature's feasibility, we validated the predictive power in two Gene Expression Omnibus (GEO) data sets. We also evaluated the correlation between the IRG signature and tumor‐infiltrating immune cells.

## 2 MATERIAL AND METHODS

### 2.1 TCGA ACC and GTEx data sets

The TCGA ACC data set provided ACC specimens (*n* = 79), whereas the GTEx data set provided normal adrenal specimens (*n* = 258). We acquired the RNA‐sequencing (RNA‐seq) data and clinical samples from the GTEx (http://www.gtexportal.org/home/index.html) and TCGA (http://portal.gdc.cancer.gov/) databases. Differentially expressed genes (DEGs) were aberrated transcripts identified under different biological context through RNA‐seq analysis.^15^ DEGs makes RNA‐seq analysis possible to get a deep understanding of pathogenesis‐related molecular mechanisms and biological functions. Therefore, differential analysis has been regarded as valuable for diagnostics, prognostics, and therapeutics of tumors. To identify DEGs from the ACC and normal adrenal samples, we used the edgeR R package.^16^ The cutoff criteria were |log Fold Change| ≥ 1 and an adjusted *p* (adj. *p*) < .05.

### 2.2 Gene set enrichment analysis (GSEA) and identification of immune‐related genes

GSEA (http://www.broadinstitute.org/gsea/index.jsp) was applied to reveal the potential functions of DEGs. Gene set permutations were performed 1000 times for each analysis. *p* < .05 was established as the cutoff point for Gene Ontology (GO) enrichments in the GSEA. We search for IRGs from the GSEA v4.2.3 (http://www.gsea-msigdb.org/gsea/downloads.jsp: Immune system process M13664, Immune response M19817)^17^ and identified 332 IRGs.

### 2.3 Identification of an immune‐related gene prognostic signature

We construct the IRG signature by using the survival R package. The identification and verification criteria for IRG signatures in the ACC specimens were as follows: (1) intact information regarding the IRG expression and clinical features (age, gender, tumor TNM stage, and survival time); and (2) specimens with total survival <30 days were excluded (some nonneoplastic factors such as severe infection or hemorrhage may have destroyed these specimens). From these, we selected 77 ACC samples for the IRG signature construction. The 77 specimens were randomly divided into the training (*n* = 39) and the testing (*n* = 38) cohorts. The training cohort was utilized for the IRG signature construction. The other cohort was utilized for validation of the IRG signature.

Next, we screened for prognosis‐related IRGs (*p* < .01), regardless of whether the IRGs were protective or deleterious according to hazard ratio (HR) as well as 95% confidence interval (CI). The 332 IRGs were analyzed by univariate Cox regression analysis in the training cohort. After that, we selected an optimal model from the prognosis‐related IRGs using the Akaike information criteria (AIC) method. The three IRGs with minimum AIC values were finally selected for the prediction model construction.

We analyzed the prognosis‐related IRGs using multivariate Cox regression analyses to construct a prognostic IRG signature and identify the coefficients.^18^, ^19^, ^20^ The following in‐house formula was used to calculate the risk score of the prognostic IGR signal for each specimen: Risk score = Expression_IRG1_ × Coefficient_IRG1_ + Expression_IRG2_ × Coefficient_IRG2_ + … + Expression_IRGn_ × Coefficient_IRGn_. The risk score of the prognostic IRG signature may be determined based on a linear combination of the IRG expression level weighted by the regression coefficients. We acquired the coefficient by log‐transformed HR, derived from the multivariate Cox regression analysis.^21^, ^22^ We divided the specimens into the low‐risk and high‐risk groups based on the median value of the risk score. Univariate and multivariate Cox regression analyses, time‐dependent receiver operating characteristic (ROC) curve, and Kaplan–Meier (KM) survival curve were conducted to evaluate the predictive value of the risk score in the training cohort.

### 2.4 Internal validation and external validation of the IRG signature

To further validate the predictive ability of the IRG signature, we used the testing cohort and the entire TCGA ACC cohort to perform internal validation, and used GSE10927 and GSE19750 cohorts from GEO to perform external validation. The validation was conducted by following the same analysis methods of the training cohort.

### 2.5 Functional enrichment analysis between different risk groups and correlation analysis

Principal component analysis (PCA) was carried out to profile the expression patterns of the low‐risk and high‐risk groups according to the IRG signature by using scatterplot3d R package. We identified DEGs in both groups and adopted the clusterProfiler and GOplot R packages for GO and Kyoto Encyclopedia of Genes and Genomes (KEGG) pathway enrichment analyses^23^ to uncover the potential capacities in the two risk groups and the TCGA ACC cohort. Furthermore, the adj. *p* < .05 was used as the cutoff value. We investigated the pairwise gene correlation of three IRGs (Inhibin subunit βA [*INHBA*], Helicase, lymphoid specific [*HELLS*], and Histone deacetylase 4 [*HDAC4*]) by adopting the Pearson correlation analysis. The *R* was determined. *p* < .05 was established as the cutoff value.

### 2.6 Tumor‐infiltrating immune cells of the three‐IRG signature

We retrieved 22 types of tumor‐infiltrating immune cells from CIBERSORT, a web tool for analyzing tumor‐infiltrating immune cells (http://cibersort.stanford.edu/).^24^, ^25^ To identify the differences between the low‐risk and high‐risk groups, we measured the quantity of each immune cell. *p* < .05 was set as the cutoff value.

### 2.7 Statistical analysis

Statistical analysis was carried out by GraphPad Prism 7.0 and R software (v3.5.3: http://www.r-project.org). The RNA‐seq data were log2‐transformed. To assess the influences of the IRG prognostic signature on OS and other clinical features, Cox regression analyses, log‐rank test, and KM method were used. The *χ* ^2^ test and Student's *t* test were adopted to measure qualitative variables and quantitative variables, respectively. According to the IRG signature and other indexes, we evaluated the predictive value using time‐dependent ROC. The cutoff value of the two‐sided *p* was set at .05 (two‐sided *p* < .05).

## 3 RESULTS

**Figure 1:**
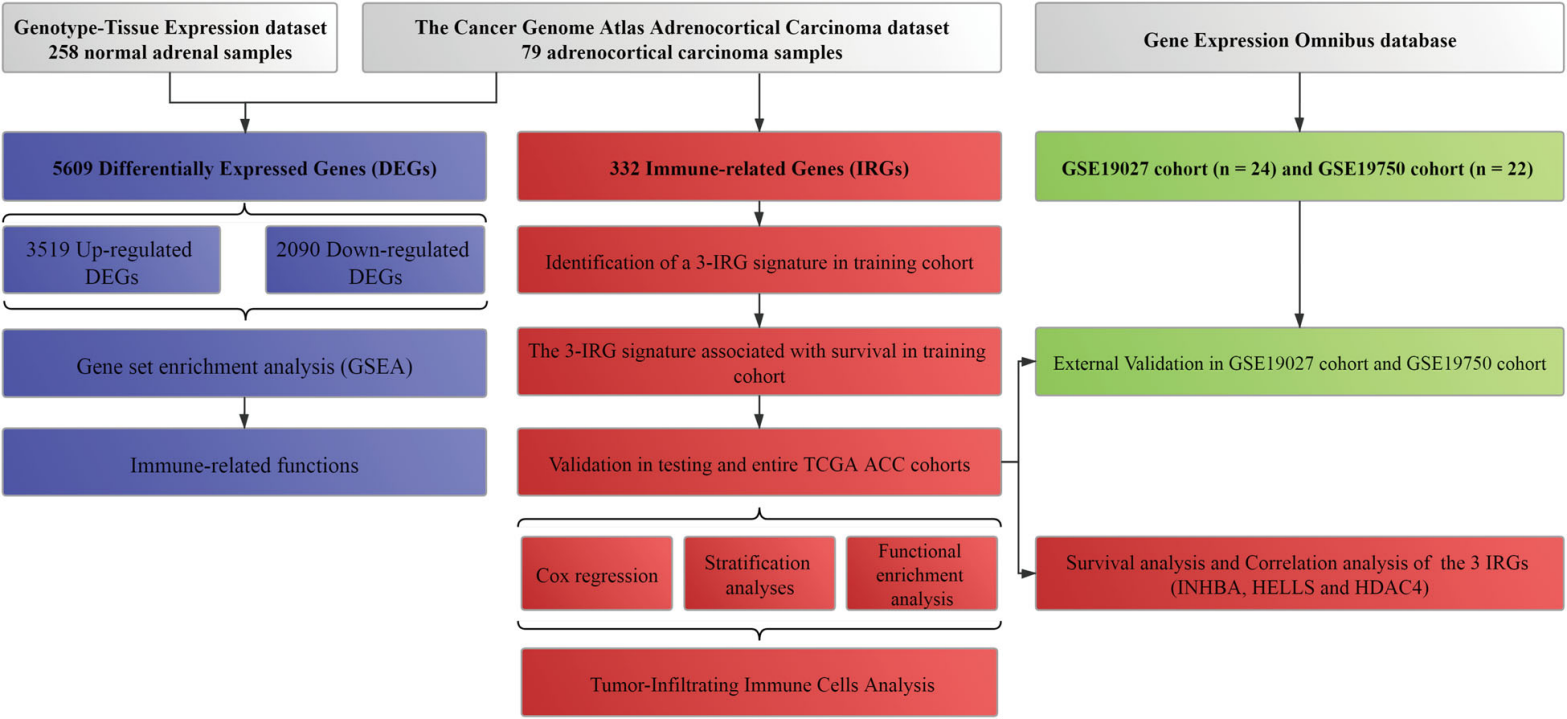
Workflow of this study. The study was carried out in The Cancer Genome Atlas (TCGA) adrenocortical carcinoma (ACC), Genotype‐Tissue Expression (GTEx), and Gene Expression Omnibus (GEO) data sets. Differentially expressed genes (DEGs) were calculated between ACC samples from TCGA ACC data set and normal adrenal samples from GTEx data set. GSEA was conducted based on all DEGs and we found the DEGs were enriched in immune‐related functions. Then, 322 immune‐associated genes (IRGs) were extracted from Molecular Signatures Database v4.0. The training cohort was used to identify prognostic IRGs and establish a prognostic signature based on three IRGs (*INHBA*, *HELLS*, and *HDAC4*). The prognosis analysis was validated in the testing cohort and the entire TCGA ACC cohort, respectively. In addition, external validation was carried out based on GSE10927 cohort and GSE19750 cohort. Tumor‐infiltrating immune cells analysis was performed based on CIBERSORT tool to investigate the association between the three‐IRG signature and immune system.

Figure 1 shows the study flowchart.

### 3.1 Identification of DEGs and functional enrichment analysis

**Figure 2:**
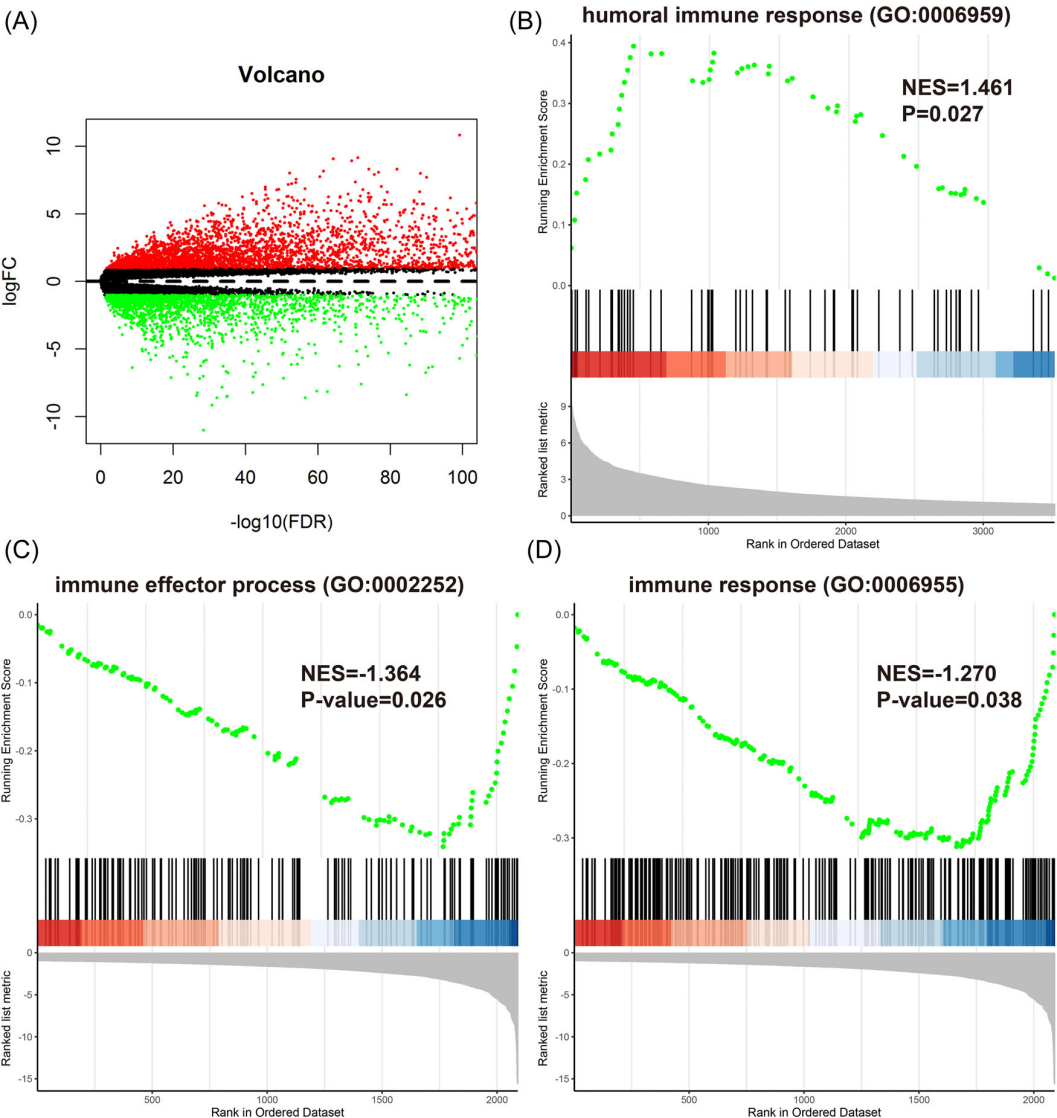
Identification of differentially expressed genes (DEGs) and gene set enrichment analysis (GSEA) between adrenocortical carcinoma (ACC) samples and normal adrenal samples. (A) Volcano plot of all DEGs. (B) Upregulated DEGs were enriched in humoral immune response. (C, D) Downregulated DEGs were enriched in immune effector process and immune response.

All genes from the TCGA ACC and GTEx data sets were analyzed via gene differential expression analysis. From these, we selected 79 ACC specimens and 258 normal adrenal specimens. We used edgeR R package to identify DEGs between ACC and normal adrenal samples to further explore the immune‐related DEGs and immune‐related functions. In total, we identified 5609 DEGs from these specimens, including 3519 upregulated and 2090 downregulated DEGs (Figure 2A). The biological capacities of DEGs determined via GO enrichment analysis was identified by GSEA. The results indicated that upregulated genes were involved in the humoral immune response (GO:0006959; *p* = .027; Figure 2B). Moreover, downregulated genes correlated with the effector process of the immune system (GO:0002252; *p* = .026) and immune response (GO:0006955; *p* = .038; Figure 2C,D), which showed that the tumorigenesis and progress of ACC are related to the immune system and immune responses.

### 3.2 Identification of immune‐related genes and the clinical characteristics of TCGA patients with ACC

We identified and obtained 332 IRGs from the Molecular Signatures Database v4.0. To construct and validate the IRG signature, 77 patients with ACC were included. These patients were randomly divided into the training (*n* = 39) and the testing (*n* = 38) cohorts. Table 1 was the clinical features of 77 ACC patients.

**Table 1:** Clinical characteristics of 77 ACC patients involved in identification and validation of the immune‐related gene prognostic signature

Characteristics	Entire TCGA ACC cohort (*N* = 77)	Detailed data		*p* a
		Training cohort (*N* = 39)	Testing cohort (*N* = 38)	
Age at diagnosis (years)				.737
≤60	60 (77.9%)	31 (79.5%)	29 (76.3%)	
>60	17 (22.1%)	8 (20.5%)	9 (23.7%)	
Gender				.206
Male	29 (37.7%)	12 (30.8%)	17 (44.7%)	
Female	48 (62.3%)	27 (69.2%)	21 (55.3%)	
Tumor stage				.809
Stage I	9 (11.7%)	6 (15.4%)	3 (7.8%)	
Stage II	37 (48.1%)	18 (46.2%)	19 (50.0%)	
Stage III	16 (20.8%)	8 (20.5%)	8 (21.1%)	
Stage IV	15 (19.4%)	7 (17.9%)	8 (21.1%)	

Abbreviations: ACC, adrenocortical carcinoma; TCGA, The Cancer Genome Atlas.

^a^ *χ* ^2^ test.

### 3.3 Identification of the IRG prognostic signature in the training cohort

We identified prognostic IRGs from the 262 IRGs in the training cohort using univariate Cox regression analysis. There were seven prognosis‐related IRGs. To construct an IRG signature, a multivariate Cox regression model with the smallest AIC was applied to the seven prognostic IRGs. Ultimately, three IRGs were selected and we constructed the three‐IRG prognostic signature. Table 2 summarizes the details relating to the univariate Cox regression analysis, descriptions, coefficients, Ensembl IDs, and gene symbols. All the three IRGs (*INHBA*, *HELLS*, and *HDAC4*) were deleterious IRGs. For all three IRGs, the univariate Cox HR was greater than 1, which suggested that patients with high expression of the three IRGs may have a shorter survival time. The following formula was established to calculate the risk score of the three‐IRG signature based on the expression of the three IRGs for OS prediction: Risk score = (1.647 × Expression_INHBA_) + (2.583 × Expression_HELLS_) + (2.61 × Expression_HDAC4_).

**Table 2:** The three immune‐related genes identified from Cox regression analysis

Gene symbol	Ensembl ID	Description	Coefficient	Univariate Cox regression analysis		
				HR	95% CI	*p*
*INHBA*	ENSG00000122641	Inhibin subunit βA	1.647	5.193	1.111–24.262	0.006
*HELLS*	ENSG00000119969	Helicase, lymphoid specific	2.583	9.882	1.956–49.925	<0.001
*HDAC4*	ENSG00000068024	Histone deacetylase 4	2.610	13.242	2.919–60.073	0.002

Abbreviations: CI, confidence interval; HR, hazard ratio.

### 3.4 Assessing the predictive capacity of the three‐IRG prognostic signature for patients with ACC in the training cohort

**Figure 3:**
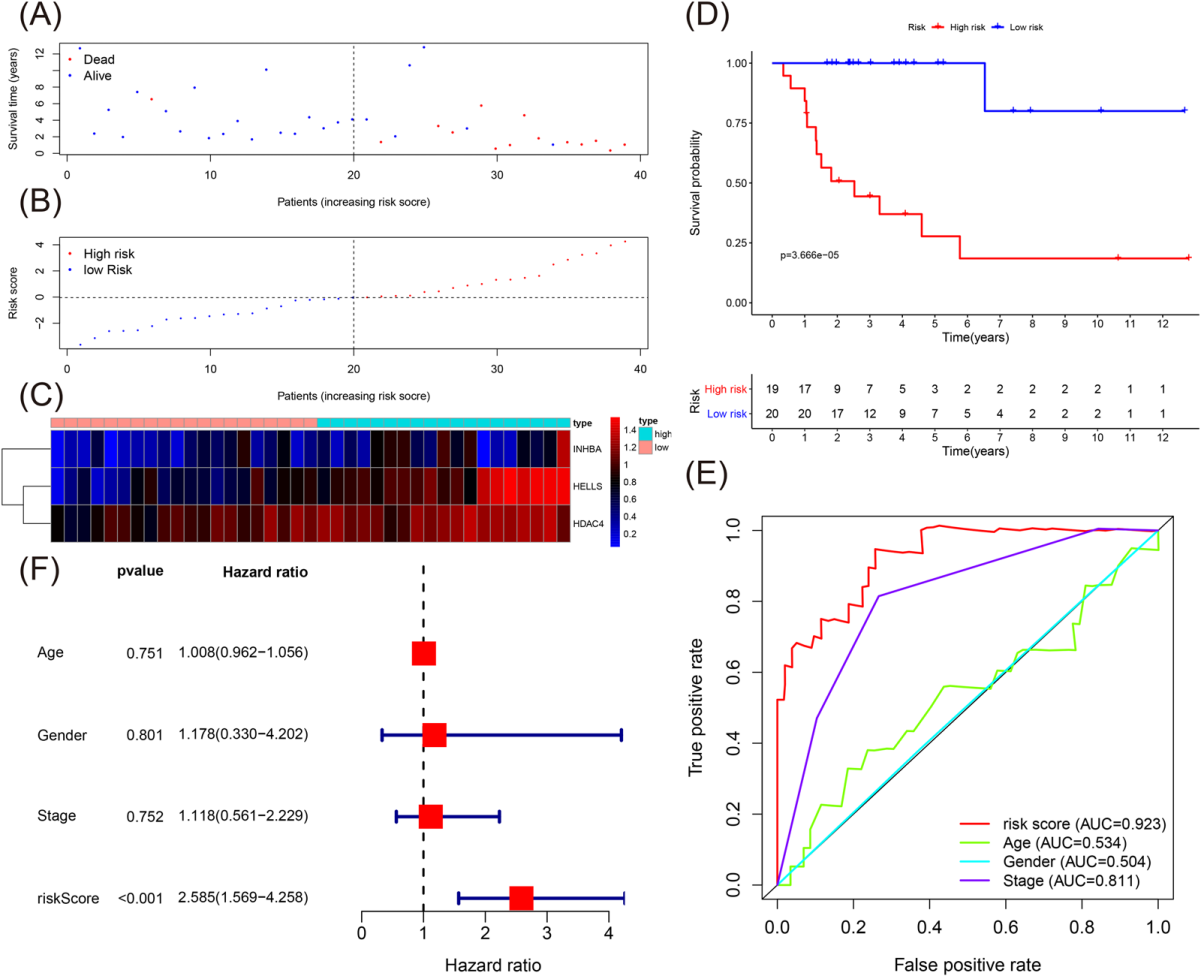
Evaluating the predictive power of the immune‐related signature in the training cohort. (A–C) Distribution of survival status, risk score, and gene expression of patients in the training cohort. (D) Kaplan–Meier survival curve of the high‐risk and low‐risk groups in the training cohort. (E) Time‐dependent receiver operating characteristic (ROC) curves and area under the ROC curve (AUC) based on the training cohort for 5‐year overall survival. (F) Forest plot for multivariate Cox regression analysis.

From the training cohort, 39 patients with ACC patients were divided into two groups based on the median value of the three‐IRG signature risk score, namely, the high‐risk (*n* = 19) and low‐risk (*n* = 20) groups. Figure 3A,B summarizes the details of each patient in the training cohort, including survival time, survival status, and the risk score. Figure 3C illustrates the expression of the three IRGs. The KM survival curve of all the patients with ACC in the training cohort is shown in Figure 3D. We analyzed the survival time and found that patients in the low‐risk group had a longer survival time than those in the high‐risk group (*p* < .001). As shown in Figure 3E, by plotting time‐dependent ROC curves of the three‐IRG prognostic signature and other clinical features in the training cohort, we identified that the area under the ROC curve (AUC) value was 0.923. We also evaluated the age, three‐IRG prognostic signature, gender, and tumor stage using multivariate Cox regression analyses, as shown in Figure 3F. From the risk score (HR = 2.585, 95% CI = 1.569–4.258, *p* < .001) of the three‐IRG signature, a unique prognostic target of overall poor survival was identified (Table 3).

**Table 3:** Univariate and multivariate Cox regression analyses of the training, testing, entire TCGA ACC, GSE10927, and GSE19750 cohorts

Variables	Univariate analysis			Multivariate analysis		
	HR	95% CI	*p*	HR	95% CI	*p*
Training cohort						
Three‐IRG signature risk score	1.003	0.968–1.040	.866	1.008	0.962–1.056	.751
Age at diagnosis	1.505	0.496–4.563	.470	1.178	0.330–4.202	.801
Gender	**2.579**	**1.441–4.617**	**.001**	1.118	0.561–2.229	.752
Tumor stage	**2.718**	**1.752–4.216**	**<.001**	**2.585**	**1.569–4.258**	**<.001**
Testing cohort						
Three‐IRG signature risk score	1.029	0.984–1.076	.209	**1.067**	**1.002–1.137**	**.044**
Age at diagnosis	0.907	0.288–2.862	.868	0.847	0.1750–4.103	.837
Gender	**3.499**	**1.700–7.202**	**.001**	**3.434**	**1.264–9.329**	**.016**
Tumor stage	**1.932**	**1.372–2.720**	**<.001**	**2.168**	**1.281–3.668**	**.004**
Entire TCGA ACC cohort						
Three‐IRG signature risk score	1.010	0.985–1.036	.430	1.019	0.987–1.052	.236
Age at diagnosis	1.043	0.473–2.299	.917	0.863	0.353–2.112	.747
Gender	**2.903**	**1.844–4.569**	**<.001**	**1.707**	**1.049–2.780**	**.031**
Tumor stage	**1.920**	**1.562–2.360**	**<.001**	**1.842**	**1.471–2.307**	**<.001**
GSE10927 cohort						
Three‐IRG signature risk score	1.012	0.977–1.048	.519	0.992	0.9589–1.026	.632
Age at diagnosis	1.471	0.510–4.242	.475	1.251	0.326–4.802	.745
Gender	**1.818**	**1.095–3.019**	**.021**	**2.330**	**1.298–4.184**	**.005**
Tumor stage	**1.863**	**1.143–3.036**	**.013**	**2.660**	**1.345–5.261**	**.005**
GSE19750 cohort						
Three‐IRG signature risk score	1.035	0.994–1.079	.097	1.019	0.978–1.063	.368
Age at diagnosis	**0.294**	**0.091–0.956**	**.042**	0.905	0.210–3.900	.894
Gender	1.143	0.700–1.865	.593	1.284	0.737–2.234	.377
Tumor stage	**1.008**	**1.003–1.013**	**<.001**	**1.008**	**1.003–1.013**	**.001**

*Note*: Bold values indicat statistically significant results.

Abbreviations: ACC, adrenocortical carcinoma; CI, confidence interval; HR, hazard ratio; IRG, immune‐associated gene; TCGA, The Cancer Genome Atlas.

### 3.5 Authentication of the three‐IRG prognostic signature in the entire TCGA ACC cohort and the testing cohort

**Figure 4:**
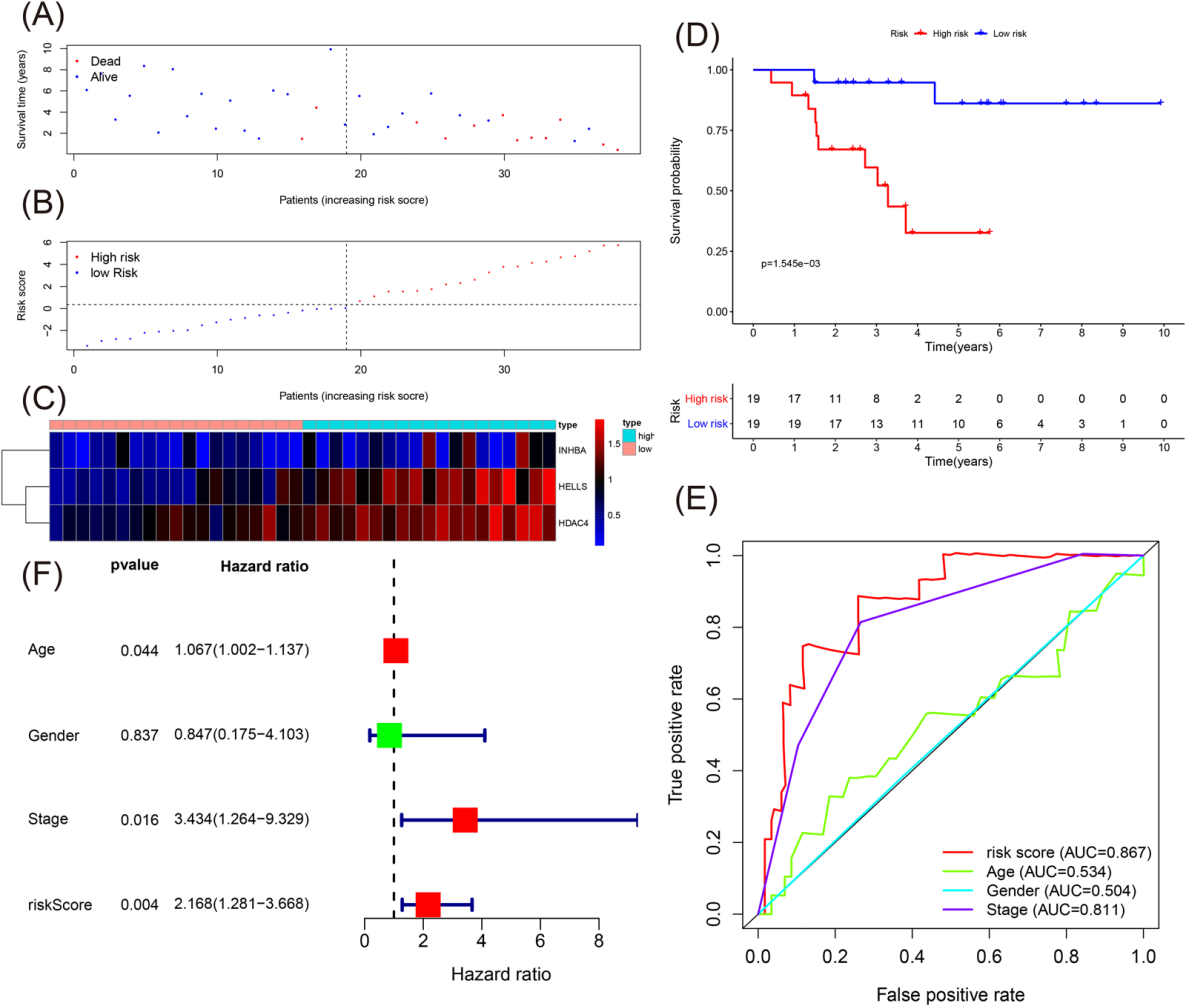
Evaluating the predictive power of the immune‐related signature in the testing cohort. (A–C) Distribution of survival status, risk score, and gene expression of patients in the testing cohort. (D) Kaplan–Meier survival curve of the high‐risk and low‐risk groups in the testing cohort. (E) Time‐dependent receiver operating characteristic (ROC) curves and area under the ROC curve (AUC) based on the testing cohort for 5‐year overall survival. (F) Forest plot for multivariate Cox regression analysis.

We utilize the three‐IRG signature to evaluate the OS of patients with ACC in the testing cohort to prove the stability and predictive capacity of the three‐IRG signature. Risk scores of the three‐IRG signature in 38 patients with ACC from the testing cohort were computed using the previously mentioned formula. The 38 ACC patients were divided into the high‐risk and low‐risk groups (*n* = 19 per group) based on the median value. Figure 4A‐B provides the clinical features of the testing cohort, the survival time, survival status, and the risk score. The expression of the three IRGs is shown in Figure 4C.

Figure 4D displays the KM survival curve of the testing cohort. Our results found that patients of the low‐risk group subsisted for a longer time compared with the high‐risk group (*p* = .002). Figure 4E shows the plotting time‐dependent ROC curves of the three‐IRG prognostic signature and other clinical features in the training cohort, which demonstrated that the AUC of the three‐IRG prognostic signature was distinctly better than other clinical features (risk value was 0.867). The results between the training cohort and the testing cohort were the same. From the multivariate Cox regression analysis, the risk score (HR = 2.168, 95% CI = 1.281–3.668, *p* = .004) confirmed the three‐IRG signature as a unique prognostic target (Figure 4F and Table 3).

**Figure 5:**
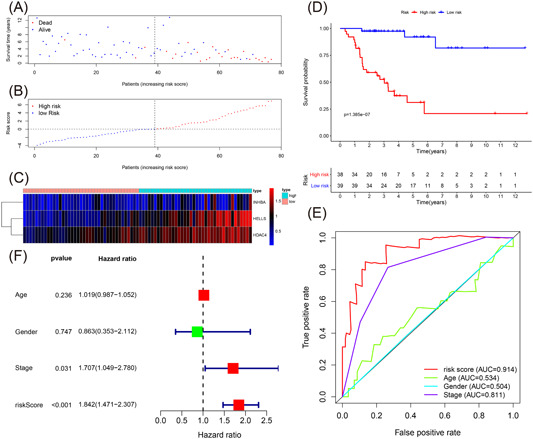
Evaluating the predictive power of the immune‐related signature in the entire The Cancer Genome Atlas (TCGA) adrenocortical carcinoma (ACC) cohort. (A–C) Distribution of survival status, risk score, and gene expression of patients in the entire TCGA ACC cohort. (D) Kaplan–Meier survival curve of the high‐risk and low‐risk groups in the entire TCGA ACC cohort. (E) Time‐dependent receiver operating characteristic (ROC) curves and area under the ROC curve (AUC) based on the entire TCGA ACC cohort for 5‐year overall survival. (F) Forest plot for multivariate Cox regression analysis.

All the patients in the TCGA ACC cohort were analyzed using time‐dependent ROC curves, KM survival curve, and multivariate Cox regression analysis. The results are shown in Figure 5 and Table 3.

### 3.6 External validation using the GSE10927 and GSE19750 cohorts

**Figure 6:**
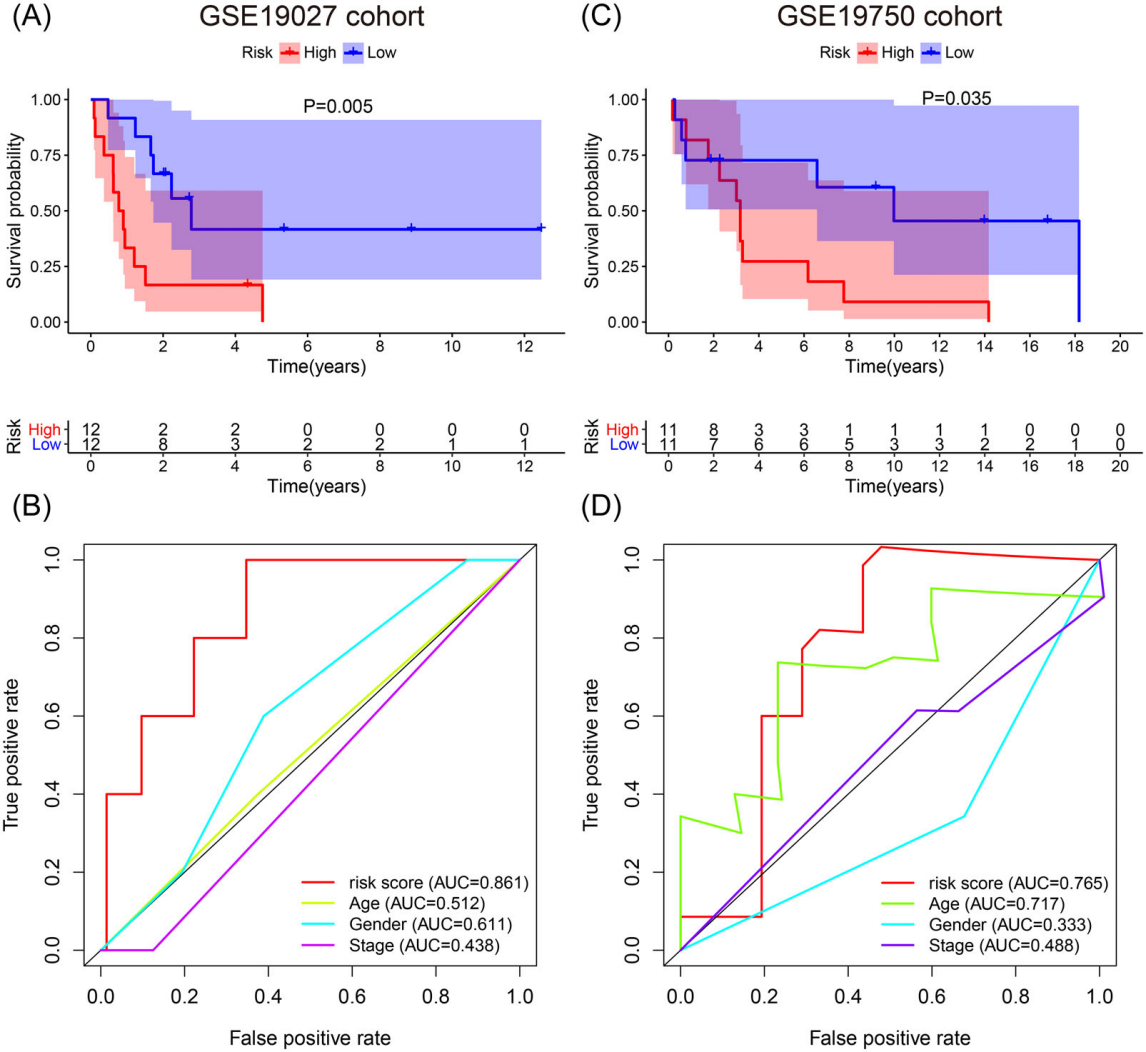
External validation for evaluating the predictive power of the immune‐related signature in GSE10927 cohort and GSE19750 cohort. (A) Kaplan–Meier survival curve of the high‐risk and low‐risk groups in GSE10927 cohort. (B) Time‐dependent receiver operating characteristic (ROC) curves and area under the ROC curve (AUC) based on GSE10927 cohort for 5‐year overall survival. (C) Kaplan–Meier survival curve of the high‐risk and low‐risk groups in GSE19750 cohort. (D) Time‐dependent ROC curves and AUC based on GSE19750 cohort for 5‐year overall survival.

To externally validate the three‐IRG signature in the GSE10927 (*n* = 24) and GSE19750 (*n* = 22) cohorts, we applied the time‐dependent ROC curve (Table 4), univariate and multivariate Cox regression analyses, and KM survival curve (Table 3). The KM survival curve and time‐dependent ROC curve for the GSE10927 cohort are shown in Figure 6A,B. The results confirmed that patients of the low‐risk group subsisted for a longer time compared to those of the high‐risk group (*p* = .005). The AUC of three‐IRG prognostic signature was distinctly better than other clinical features (risk value was 0.861). Similar results were found in the GSE19750 cohort (Figure 6C,D).

**Table 4:** AUCs from time‐dependent ROC curves

Variables	Entire TCGA ACC cohort	GSE10927 cohort	GSE19750 cohort
Three‐IRG signature risk score			
1‐Year	0.883	0.853	0.792
3‐Year	0.923	0.956	0.897
5‐Year	0.914	0.861	0.765
Age at diagnosis			
1‐Year	0.607	0.537	0.607
3‐Year	0.534	0.536	0.643
5‐Year	0.534	0.512	0.717
Gender			
1‐Year	0.512	0.694	0.512
3‐Year	0.504	0.548	0.480
5‐Year	0.504	0.611	0.333
Tumor stage			
1‐Year	0.611	0.616	0.611
3‐Year	0.811	0.791	0.676
5‐Year	0.811	0.438	0.488

Abbreviations: ACC, adrenocortical carcinoma; AUC, area under the ROC curve; ROC, receiver operating characteristic; TCGA, The Cancer Genome Atlas.

### 3.7 Survival, clinical features, and the three‐IRG prognostic signature

**Figure 7:**
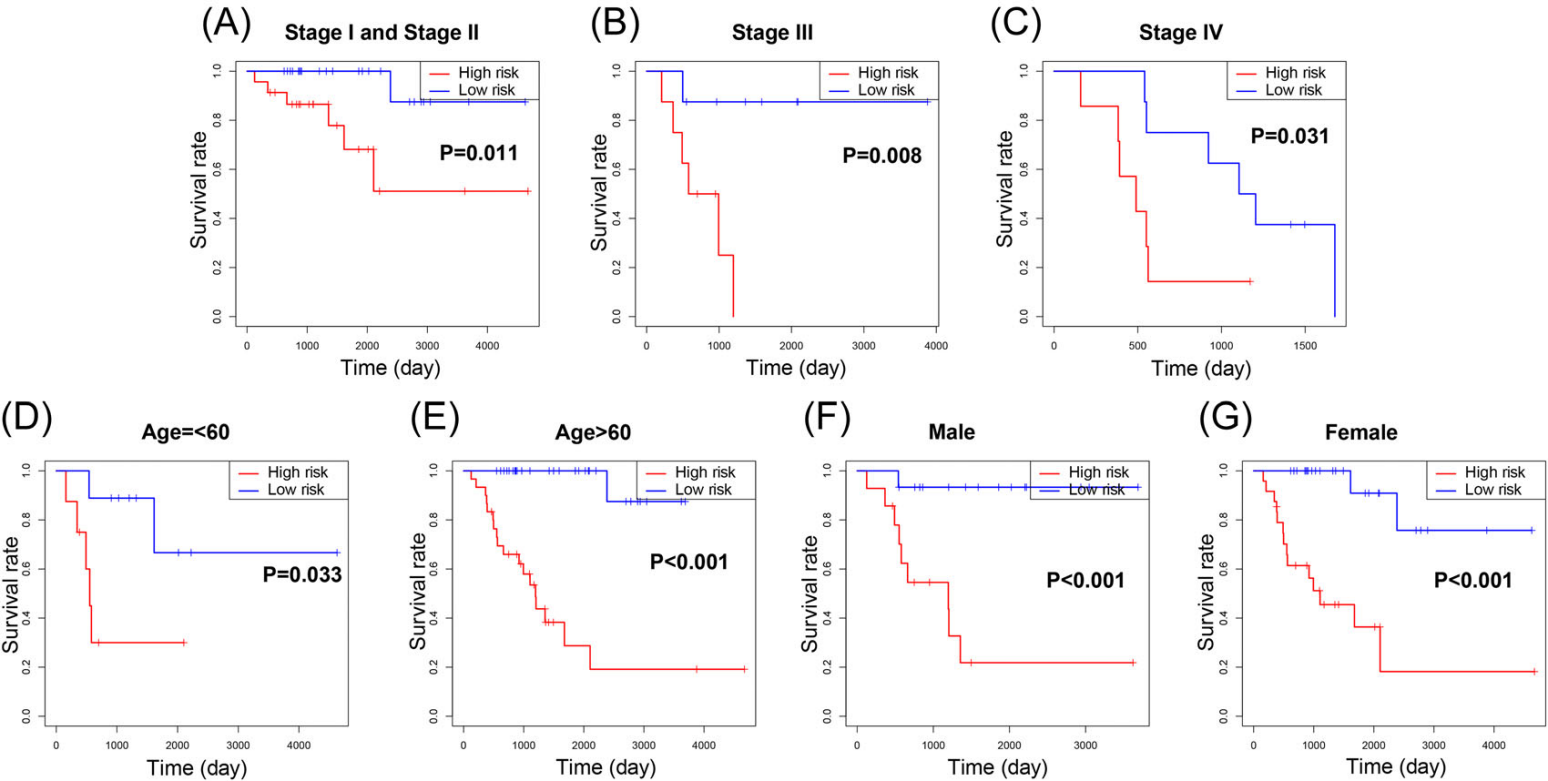
Stratified survival analyses with immune‐related prognostic signature in the entire The Cancer Genome Atlas (TCGA) adrenocortical carcinoma (ACC) cohort. (A–G) Kaplan–Meier overall survival (OS) curves in subgroups stratified by different clinical characteristics.

We analyzed the entire TCGA ACC cohort by stratified survival analysis, to further verify the prognostic capacity and explore the extensive feasibility of the three‐IRG signature (Figure 7A–G). The samples with Stage I–II (*p* = .011), Stage III (*p* = .008), and Stage IV (*p* = .031) were assigned to the low‐risk group, which had better survival than the high‐risk group. Similar results were found in diverse ages and different gender.

**Figure 8:**
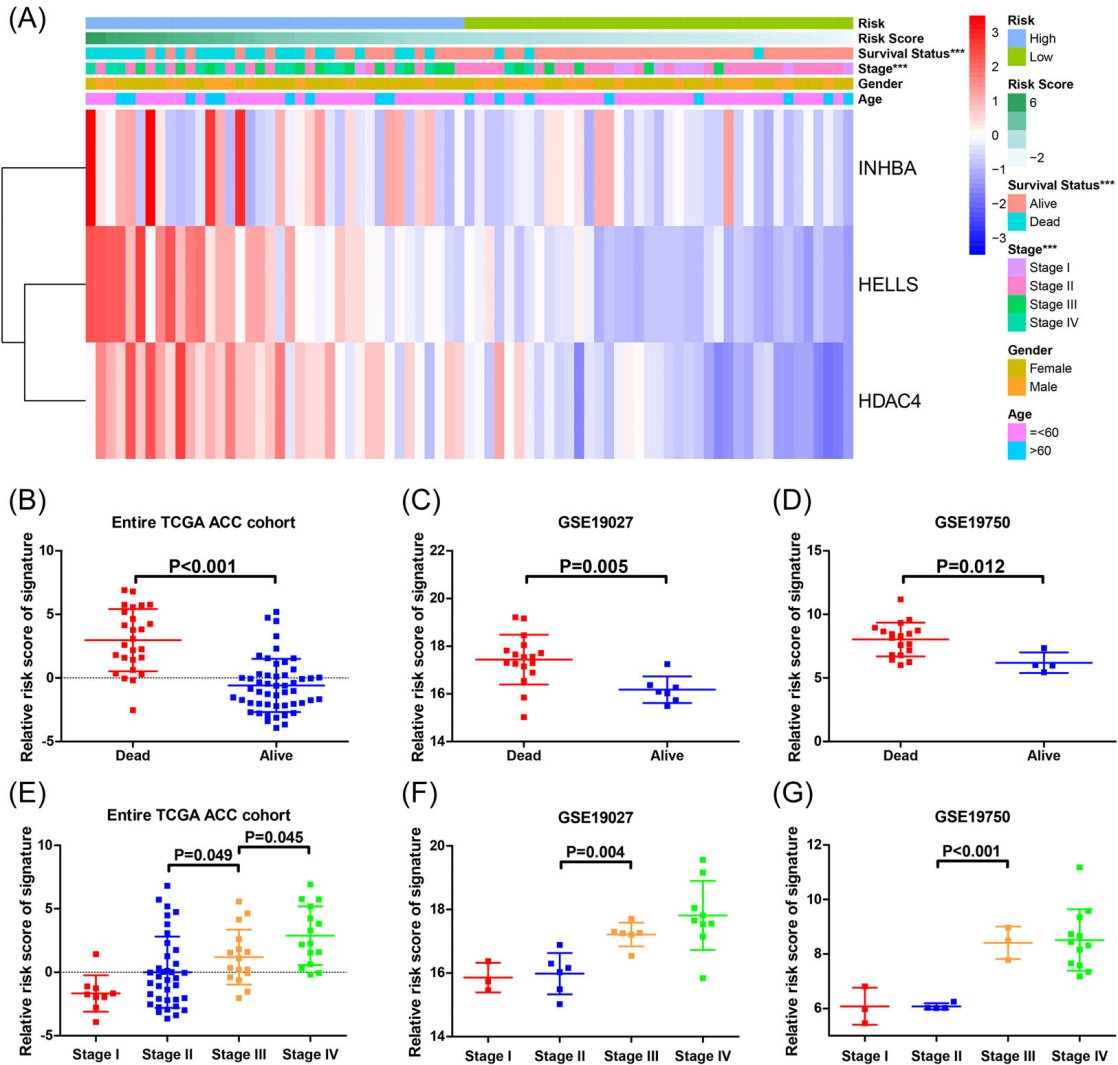
Association between clinical characteristics and the immune‐related prognostic signature. (A) Heat map for distribution of clinicopathologic features, and gene expression in low‐risk and high‐risk groups in the entire The Cancer Genome Atlas (TCGA) adrenocortical carcinoma (ACC) cohort. (B–D) Risk score comparison between alive and dead patients. (E–G) Risk score comparison between different tumor stages. ***p < .005.

Clinical features were distributed along with the three‐IRG prognostic signature. The high‐risk group had a higher number of mortalities, which was reflective of poorer survival in these patients who had a high‐risk value (*p* < .05) (Figure 8B–D). Furthermore, there were different risk scores for patients in different disease stages. Patients at the inchoate stages (Stage I and Stage II) have lower risk values than those at more advanced stages (Stage III and Stage IV; *p* < .05; Figure 8E–G), which verified that risk scores of the IRG prognostic signature were distinctly related to the development of ACC.

### 3.8 PCA and functional enrichment analysis

**Figure 9:**
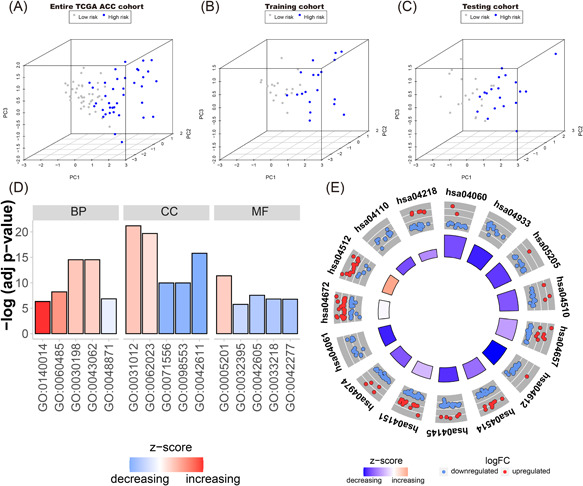
Principal component analysis (PCA) and functional enrichment analyses. PCA based on the three IRGs indicated low‐risk and high‐risk groups were generally distributed in two different directions in (A) TCGA ACC cohort, (B) the training cohort, and (C) the testing cohort, respectively. (D) Gene ontology (GO) functional enrichment analysis between low‐risk and high‐risk groups based on TCGA ACC cohort. (E) Kyoto Encyclopedia of Genes and Genomes (KEGG) pathway enrichment analysis between low‐risk and high‐risk groups based on TCGA ACC cohort.

To determine the disparate distribution patterns between the high‐risk and low‐risk groups based on the three IRGs, we performed a PCA analysis. The two groups were obviously separated in two different directions based on the three IRGs. It was evident that the samples in the high‐risk group were significantly different from that of the low‐risk group (Figure 9A–C). From the entire TCGA ACC cohort, 664 upregulated and 563 downregulated DEGs were revealed between low‐risk and high‐risk groups. We identified their capacity using the KEGG and GO functional enrichment analyses. The top 15 terms of GO are listed in Figure 9D and Table 5. The top 15 terms of KEGG are shown in Figure 9E and Table 6. We found that they were significantly enriched in the extracellular matrix regulation, cell cycle, and phosphatidylinositol 3‐kinase (PI3K)‐Akt signaling pathway.

**Table 5:** GO functional enrichment analysis between high‐risk and low‐risk groups in entire TCGA ACC cohort

ID	Term	Category	Adjusted *p*
GO:0030198	Extracellular matrix organization	Biological process	2.92E − 15
GO:0043062	Extracellular structure organization	Biological Process	2.92E − 15
GO:0060485	Mesenchyme development	Biological process	6.13E − 09
GO:0048871	Multicellular organismal homeostasis	Biological process	1.40E − 07
GO:0140014	Mitotic nuclear division	Biological process	4.74E − 07
GO:0031012	Extracellular matrix	Cellular component	6.31E − 22
GO:0062023	Collagen‐containing extracellular matrix	Cellular component	2.08E − 20
GO:0042611	MHC protein complex	Cellular component	1.56E − 16
GO:0071556	Integral component of lumenal side of endoplasmic reticulum membrane	Cellular component	1.09E − 10
GO:0098553	Lumenal side of endoplasmic reticulum membrane	Cellular component	1.09E − 10
GO:0005201	Extracellular matrix structural constituent	Molecular function	4.23E − 12
GO:0042605	Peptide antigen binding	Molecular function	2.80E − 08
GO:0033218	Amide binding	Molecular function	1.52E − 07
GO:0042277	Peptide binding	Molecular function	1.67E − 07
GO:0032395	MHC class II receptor activity	Molecular function	1.75E − 06

Abbreviations: ACC, adrenocortical carcinoma; GO, Gene Ontology; MHC, major histocompatibility complex; TCGA, The Cancer Genome Atlas.

**Table 6:** KEGG pathway enrichment analysis between high‐risk and low‐risk groups in entire TCGA ACC cohort

ID	Term	Adjusted *p*
hsa04061	Viral protein interaction with cytokine and cytokine receptor	4.67E − 08
hsa04672	Intestinal immune network for IgA production	3.63E − 07
hsa04512	ECM–receptor interaction	3.63E − 07
hsa04110	Cell cycle	3.77E − 07
hsa04218	Cellular senescence	5.61E − 07
hsa04060	Cytokine–cytokine receptor interaction	1.97E − 05
hsa04933	AGE‐RAGE signaling pathway in diabetic complications	1.97E − 05
hsa05205	Proteoglycans in cancer	2.23E − 05
hsa04151	PI3K‐Akt signaling pathway	7.38E − 05
hsa04974	Protein digestion and absorption	1.09E − 04
hsa04612	Antigen processing and presentation	1.41E − 06
hsa04514	CAMs	3.53E − 06
hsa04145	Phagosome	6.89E − 06
hsa04510	Focal adhesion	1.65E − 04
hsa04657	IL‐17 signaling pathway	4.21E − 04

Abbreviations: ACC, adrenocortical carcinoma; AGE‐RAGE, advanced glycationend products‐receptor of AGES; CAMS, Cell adhesion molecules; ECM, extracellular matrix; IL‐17, interleukin‐17; KEGG, Kyoto Encyclopedia of Genes and Genome; PI3K, phosphatidylinositol 3‐kinase; TCGA, The Cancer Genome Atlas.

### 3.9 Survival analysis and correlation analysis of *INHBA*, *HELLS*, and *HDAC4*

**Figure 10:**
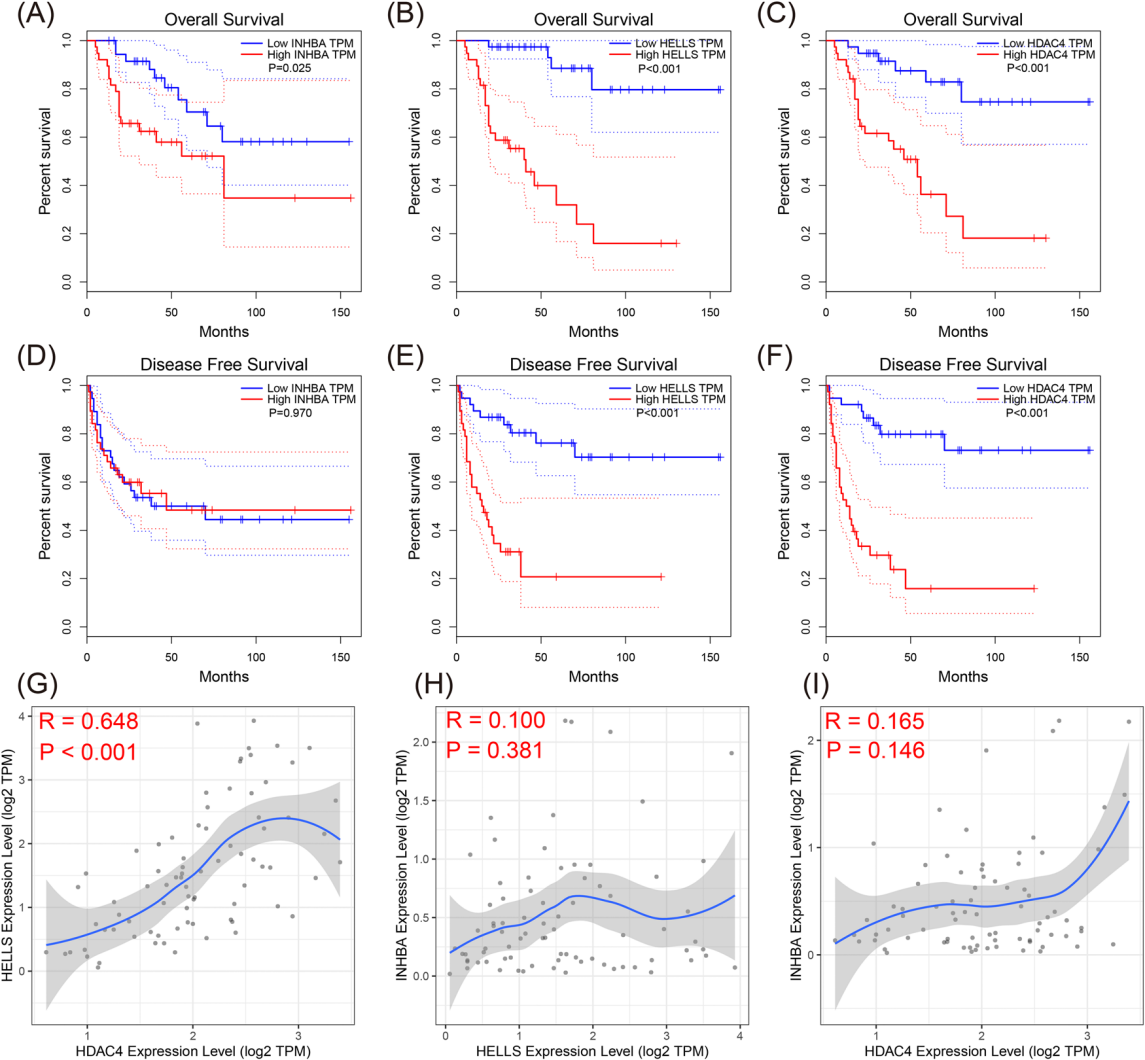
Survival analysis and correlation analysis of three immune‐related genes (*INHBA*, *HELLS*, and *HDAC4*) based on The Cancer Genome Atlas (TCGA) adrenocortical carcinoma (ACC) cohort. Overall survival (OS) for (A) Inhibin subunit βA (INHBA), (B) Helicase, lymphoid specific (HELLS), and (C) Histone deacetylase 4 (HDAC4). Disease‐free survival (DFS) for (D) INHBA, (E) HELLS, and (F) HDAC4. (G–I) Correlation analyses among INHBA, HELLS, and HDAC4.

We further explored the prognostic value of the three IRGs (*INHBA*, *HELLS*, and *HDAC4*) for construction of the signature and identified that higher expression levels of *INHBA*, *HELLS*, and *HDAC4* were related to worse OS time among patients with ACC (*p* < .05; Figure 10A–C). Higher expression levels of HELLS and HDAC4 also indicated worse disease‐free survival (Figure 10D–F). INHBA, HELLS, and HDAC4 in the TCGA ACC data set were analyzed by pairwise correlation analysis. The expression levels of HELLS and HDAC4 demonstrated an obvious positive correlation (*p* < .001 and *R* = 0.648). An increase in HELLS was related to an increase in HDAC4 (Figure 10G).

### 3.10 Correlation between tumor‐infiltrating immune cells and the three‐IRG signature

**Figure 11:**
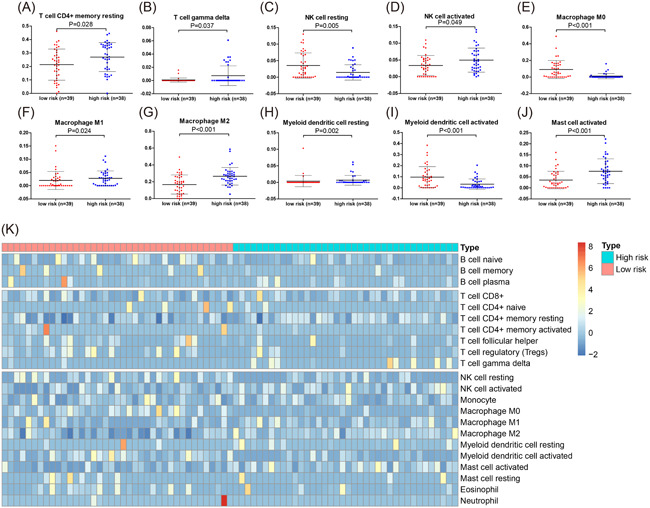
Tumor‐infiltrating immune cells analysis based on The Cancer Genome Atlas (TCGA) adrenocortical carcinoma (ACC) cohort. (A–J) Comparisons of tumor‐infiltrating immune cells between high‐risk and low‐risk groups. (K) Heat map for distribution of 22 immune cells between high‐risk and low‐risk groups.

**Figure 12:**
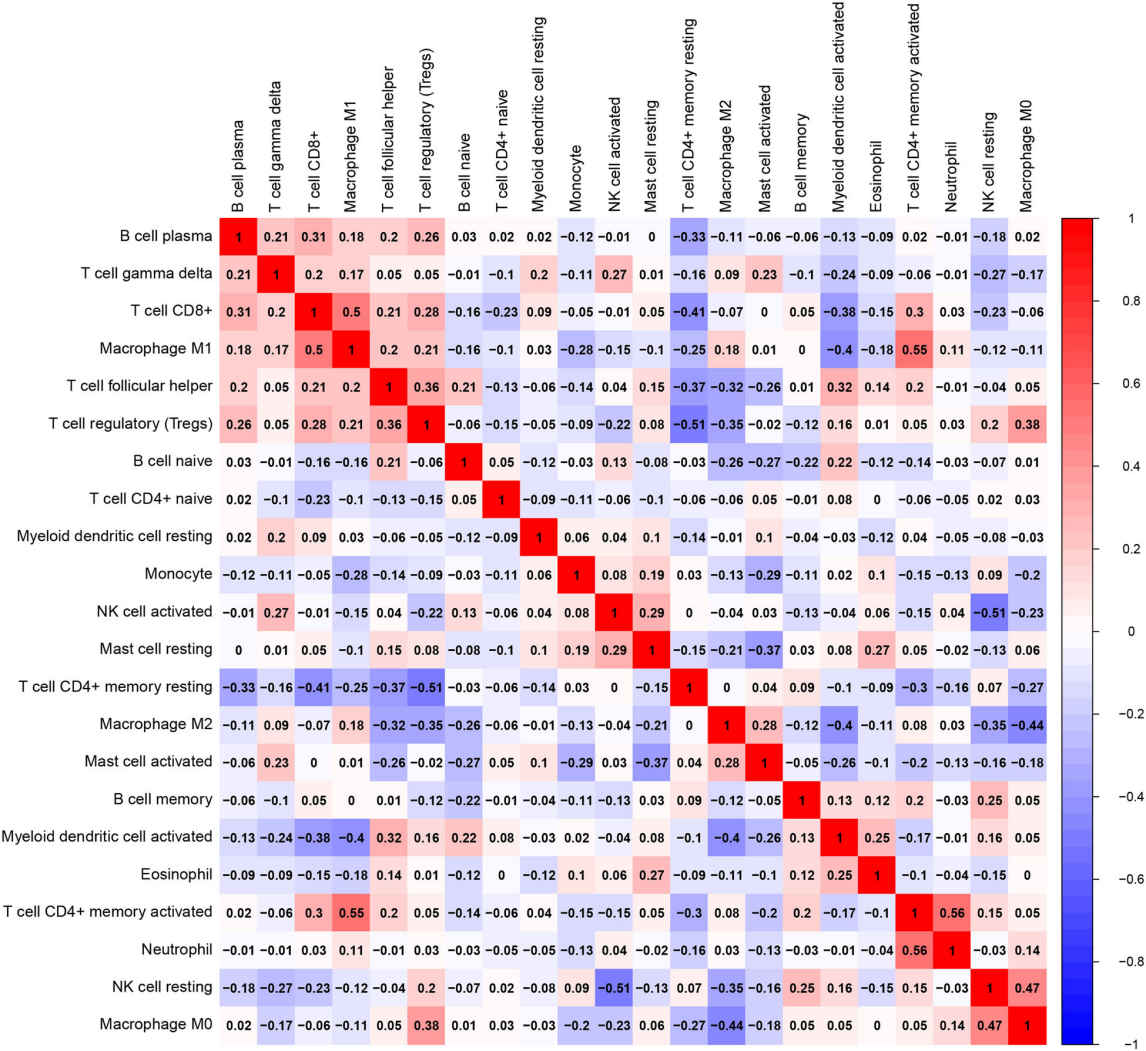
Correlation heat map of 22 immune cells in The Cancer Genome Atlas (TCGA) adrenocortical carcinoma (ACC) cohort.

We compared the tumor‐infiltrating immune cells between low‐risk and high‐risk groups in the entire TCGA ACC cohort, to investigate the relationship between tumor immune microenvironment and the three‐IRG prognostic signature. T cells, natural killer (NK) cells, macrophage cells, mast cells, and myeloid dendritic cells showed disparate abundance in the low‐risk and high‐risk groups (*p* < .05; Figure 11). The correlation heat map was also plotted based on the distribution of immune cells in patients with ACC (Figure 12).

## 4 DISCUSSION

Accumulating evidence indicated that aberrantly expressed genes in tumors may be potential biomarkers for diagnosis, target therapy, and prognosis through bioinformatics analysis.^26^ In cancer treatment, immunotherapy is gaining more and more attention. Currently, we can identify potential prognostic targets, analyze underlying mechanisms, and explore valuable IRGs through high‐throughput sequencing data.^27^, ^28^ We researched IRGs using the TCGA ACC data set and constructed a three‐IRG prognostic signature that may be used as a prognostic indicator or immune therapeutic target in patients with ACC.

The immune system impacts the tumor microenvironment, particularly tumor immune escape.^26^ Immune components have quite a significant influence on the clinical outcomes and gene expression by tumor tissues, which exist in the tumor microenvironment.^9^, ^29^, ^30^ There were 3519 upregulated and 2090 downregulated DEGs found in ACC samples and normal adrenal samples in this study. GSEA suggested that these DEGs are involved in the humoral immune response (GO:0006959), and the immune effector process (GO:0002252) and immune response (GO:0006955), which indicated that the development of ACC is significantly correlated with immune responses and the immune system (Figure 2).

We extracted 332 IRGs and constructed an IRG signature based on three IRGs (*INHBA*, *HELLS*, and *HDAC4*) in the training cohort. Based on the median value of the three‐IRG signature risk score, we divided the samples into the low‐risk and high‐risk groups. We discovered that the prognosis of samples could be distinguished by the three‐IRG signature. The OS of the high‐risk group was worse than those of the low‐risk group (Figure 3D). The multivariate Cox regression analysis suggested that the risk score may be regarded as a unique prognostic target of OS through multivariate Cox regression analyses and the three‐IRG signature was superior to other clinicopathologic features according to the ROC curve. The above findings were verified the testing cohort, and the whole TCGA ACC, GSE10927, and GSE19750 cohorts.

We then explored the wide applicability of the three‐IRG signature. Survival analyses were conducted in disparate subgroups, stratified by age, tumor stage, and gender. We found that the three‐IRG signature was applicable in various subgroups (Figure 7). Moreover, we observed that the risk score of patients with early‐stage ACC (Stage II and Stage I) was lower than the advanced one (Stage IV and Stage III; Figure 8). Our findings demonstrated that the three‐IRG prognostic signature is not only a potential predictive indicator of tumor progression but can be applied to all patients with ACC.

Functional enrichment analyses demonstrated that the DEGs between low‐risk and high‐risk groups were obviously enriched in cell cycle function and PI3K‐Akt signaling pathway. Cell cycle regulation is known to play a vital role in the differentiation and growth of tumor cells.^31^ Subramanian et al.^32^ identified a series of genes involved in regulating pathways of the cell cycle and overexpression of these genes was correlated with poorer OS in patients with ACC. LncRNA‐HOTAIR participated in ACC development by regulating the cell cycle and proliferating ACC cells.^33^ PI3K‐Akt signaling pathway is a key driver in carcinogenesis and Akt overactivation has been verified in various endocrine gland neoplasms, including thyroid carcinoma subtypes, parathyroid carcinoma, pituitary tumor, and pheochromocytoma.^34^ In vitro experiments revealed that low expression of SCTR could stimulate proliferation via the PI3K/AKT signaling cascade in ACC cells.^35^

Recently, with the advent of checkpoint inhibitors and targeted immunotherapy, a greater focus has been placed on tumor immunotherapy for patients with ACC.^36^, ^37^ Our study evaluated the prognostic value of three IRGs for the construction of the IRG signature. Higher expressions of *INHBA*, *HELLS*, and *HDAC4* were correlated with worse OS time for patients with ACC. Hence, the three IRGs may be promising therapeutic targets for ACC.

INHBA encodes a member of the transforming growth factor‐β super‐family of proteins.^38^ INHBA participates in the regulation of the immune system, especially in skin suffering from repetitive ultraviolet‐B irradiation.^39^ Mesenchymal stromal cells inhibited monocytes through upregulating INHBA in patients with myelodysplastic syndrome.^40^ IDO inhibitor induced the upregulation of IHNBA in regulating the functions of NK cells among sarcoma patients.^41^

HELLS belongs to the SNF2‐family, such as chromatin remodeling proteins.^42^ HELLS mutation leads to immune deficiency syndrome in children.^43^ A study based on adult mice suggested that although HELLS is not indispensable for lymphoid development, it is still necessary for the proliferation of peripheral T lymphocytes.^44^ In addition, high‐dose‐rate γ‐irradiation could impair immune signaling pathways by inducing the downregulation of HELLS.^45^

HDAC4 is one of the histone‐modifying enzymes, which are the main epigenetic regulators in the control of inflammatory processes. Furthermore, overexpression of HDAC4 may suppress the production of Type I interferons induced by pattern‐recognition receptors in innate immunity.^46^ Aberrant epithelial responses may arise via a self‐defense mechanism generated by miR‐22 through suppressing HDAC4 expression in epithelial cells.^47^ Moreover, the HDAC4/PGRN and HDAC4/nuclear factor‐κB axes have been involved in the regulation of inflammatory cytokines in rheumatoid arthritis.^48^

To identify gene signatures and further create clinical predictive models, high‐throughput sequencing data has been utilized in large‐scale research studies. Xu et al. stated that a signature could forecast the prognosis of ACC according to survival‐associated alternative splicing events.^49^ We constructed an immune‐related signature based on 3 IRGs in the TCGA ACC data set and further validated its application in two GEO data sets. Furthermore, the predictive capacity of the signature was further confirmed. Besides, we also explored the association between tumor‐infiltrating immune cells and the three‐IRG signature and we found that T cells, NK cells, macrophage cells, myeloid dendritic cells and mast cells showed diverse abundance between the low‐risk and high‐risk groups, which further verified the immune correlation of this signature.^14^

Although the three IRGs were regarded as therapeutic targets for the treatment of ACC, the underlining mechanisms have not been clarified, which was one limitation of this study. Therefore, more in vitro and in vivo experiments based on a larger sample size are necessary for validating these findings and applying our results into clinical immunotherapy practice in the future.

## 5 CONCLUSION

We identified an immune‐related signature based on 3 IRGs in ACC. The three‐IRG signature has important clinical significance, not only as a good classifier in distinct subgroups of ACC, but also as a unique prognostic target for ACC. Inhibitors of the three novel IRGs (*INHBA*, *HELLS*, and *HDAC4*) might activate immune cells in ACC immune microenvironment and play a synergistic role in combination therapy with immunotherapy for ACC in the future.

## AUTHOR CONTRIBUTIONS

Chengdang Xu, Caipeng Qin, Yun Peng, Jingang Jian, Chengdang Xu, and Xinan Wang performed the research. Chengdang Xu, Jingang Jian, and Yuxuan Song designed the research study. Chengdang Xu, Yuxuan Song, Jingang Jian, Xi Chen, and Xinan Wang analyzed the data. Chengdang Xu and Denglong Wu contributed essential tools. Chengdang Xu, Yun Peng, Jingang Jian, Yuxuan Song, Xi Chen, Xinan Wang, and Denglong Wu wrote the paper.

## CONFLICT OF INTEREST

The authors declare no conflict of interest.

## ETHICS STATEMENT

This study does not involve relevant ethical issues.

## Data Availability

All data were extracted from public databases: TCGA ACC data set (http://portal.gdc.cancer.gov/), GTEx database (http://www.gtexportal.org/home/index.html), and GEO database (http://www.ncbi.nlm.nih.gov/geo/).

## References

[1] Else T , Kim AC , Sabolch A , et al. Adrenocortical carcinoma. Endocr Rev. 2014;35:282‐326. 24423978 10.1210/er.2013-1029 PMC3963263

[2] Fassnacht M , Dekkers OM , Else T , et al. European Society of Endocrinology Clinical Practice Guidelines on the management of adrenocortical carcinoma in adults, in collaboration with the European Network for the study of adrenal tumors. Eur J Endocrinol. 2018;179:G1‐G46. 30299884 10.1530/EJE-18-0608

[3] Fassnacht M , Libé R , Kroiss M , Allolio B . Adrenocortical carcinoma: a clinician's update. Nat Rev Endocrinol. 2011;7:323‐335. 21386792 10.1038/nrendo.2010.235

[4] Puglisi S , Perotti P , Cosentini D , et al. Decision‐making for adrenocortical carcinoma: surgical, systemic, and endocrine management options. Expert Rev Anticancer Ther. 2018;18:1125‐1133. 30117750 10.1080/14737140.2018.1510325

[5] Bellantone R , Ferrante A , Boscherini M , et al. Role of reoperation in recurrence of adrenal cortical carcinoma: results from 188 cases collected in the Italian National Registry for adrenal cortical carcinoma. Surgery. 1997;122:1212‐1218. 9426440 10.1016/s0039-6060(97)90229-4

[6] Gonzalez RJ , Tamm EP , Ng C , et al. Response to mitotane predicts outcome in patients with recurrent adrenal cortical carcinoma. Surgery. 2007;142:867‐875. 18063070 10.1016/j.surg.2007.09.006

[7] Yu WD , Wang H , He QF , Xu Y , Wang XC . Long noncoding RNAs in cancer‐immunity cycle. J Cell Physiol. 2018;233:6518‐6523. 29574911 10.1002/jcp.26568 PMC13482439

[8] Pagès F , Berger A , Camus M , et al. Effector memory T cells, early metastasis, and survival in colorectal cancer. N Engl J Med. 2006;353:2654‐2666. 10.1056/NEJMoa051424 16371631

[9] Yoshihara K , Shahmoradgoli M , Martínez E , et al. Inferring tumour purity and stromal and immune cell admixture from expression data. Nat Commun. 2013;4:2612. 24113773 10.1038/ncomms3612 PMC3826632

[10] Martinez‐Bosch N , Vinaixa J , Navarro P . Immune evasion in pancreatic cancer: from mechanisms to therapy. Cancers. 2018;10:6. 10.3390/cancers10010006 PMC5789356 29301364

[11] Kim R , Emi M , Tanabe K . Cancer immunoediting from immune surveillance to immune escape. Immunology. 2007;121:1‐14. 17386080 10.1111/j.1365-2567.2007.02587.x PMC2265921

[12] Peng Y , Song Y , Ding J , Li N , Zhang Z , Wang H . Identification of immune‐related biomarkers in adrenocortical carcinoma: immune‐related biomarkers for ACC. Int Immunopharmacol. 2020;88:106930. 32919215 10.1016/j.intimp.2020.106930

[13] Jin Y , Wang Z , He D , et al. Analysis of m6A‐related signatures in the tumor immune microenvironment and identification of clinical prognostic regulators in adrenocortical carcinoma. Front Immunol. 2021;12:637933. 33746977 10.3389/fimmu.2021.637933 PMC7966528

[14] Huang R , Liu Z , Tian T , et al. The construction and analysis of tumor‐infiltrating immune cells and ceRNA networks in metastatic adrenal cortical carcinoma. Biosci Rep. 2020;40:BSR20200049. 32175564 10.1042/BSR20200049 PMC7103591

[15] Anders S , McCarthy DJ , Chen Y , et al. Count‐based differential expression analysis of RNA sequencing data using R and Bioconductor. Nat Protoc. 2013;8:1765‐1786. 23975260 10.1038/nprot.2013.099

[16] Robinson MD , McCarthy DJ , Smyth GK . edgeR: a Bioconductor package for differential expression analysis of digital gene expression data. Bioinformatics. 2009;26:139‐140. 19910308 10.1093/bioinformatics/btp616 PMC2796818

[17] Subramanian A , Tamayo P , Mootha VK , et al. Gene set enrichment analysis: a knowledge‐based approach for interpreting genome‐wide expression profiles. Proc Natl Acad Sci USA. 2005;102:15545‐15550. 16199517 10.1073/pnas.0506580102 PMC1239896

[18] Bao ZS , Li MY , Wang JY , et al. Prognostic value of a nine‐gene signature in glioma patients based on mRNA expression profiling. CNS Neurosci Ther. 2014;20:112‐118. 24279471 10.1111/cns.12171 PMC6493176

[19] Cai J , Zhang W , Yang P , et al. Identification of a 6‐cytokine prognostic signature in patients with primary glioblastoma harboring M2 microglia/macrophage phenotype relevance. PLoS One. 2015;10:e126022. 10.1371/journal.pone.0126022 PMC4433225 25978454

[20] Zhang CB , Zhu P , Yang P , et al. Identification of high risk anaplastic gliomas by a diagnostic and prognostic signature derived from mRNA expression profiling. Oncotarget. 2015;6:36643‐36651. 26436699 10.18632/oncotarget.5421 PMC4742201

[21] Lossos IS , Czerwinski DK , Alizadeh AA , et al. Prediction of survival in diffuse large‐B‐cell lymphoma based on the expression of six genes. N Engl J Med. 2004;350:1828‐1837. 15115829 10.1056/NEJMoa032520

[22] Zhang W , Zhang J , Yan W , et al. Whole‐genome microRNA expression profiling identifies a 5‐microRNA signature as a prognostic biomarker in Chinese patients with primary glioblastoma multiforme. Cancer. 2013;119:814‐824. 22990979 10.1002/cncr.27826

[23] Yu G , Wang L , Han Y , He Q . clusterProfiler: an R package forcomparing biological themes among gene clusters. OMICS. 2012;16:284‐287. 22455463 10.1089/omi.2011.0118 PMC3339379

[24] Newman AM , Liu CL , Green MR , et al. Robust enumeration of cell subsets from tissue expression profiles. Nat Methods. 2015;12:453‐457. 25822800 10.1038/nmeth.3337 PMC4739640

[25] Newman AM , Steen CB , Liu CL , et al. Determining cell type abundance and expression from bulk tissues with digital cytometry. Nat Biotechnol. 2019;37:773‐782. 31061481 10.1038/s41587-019-0114-2 PMC6610714

[26] Cao J , Yang X , Li J , et al. Screening and identifying immune‐related cells and genes in the tumor microenvironment of bladder urothelial carcinoma: based on TCGA database and bioinformatics. Front Oncol. 2020;9:1533. 32010623 10.3389/fonc.2019.01533 PMC6974676

[27] Wang W , Zhao Z , Yang F , et al. An immune‐related lncRNA signature for patients with anaplastic gliomas. JNO. 2018;136:263‐271. 10.1007/s11060-017-2667-6 29170907

[28] Wei C , Liang Q , Li X , et al. Bioinformatics profiling utilized a nine immune‐related long noncoding RNA signature as a prognostic target for pancreatic cancer. J Cell Biochem. 2019;120:14916‐14927. 31016791 10.1002/jcb.28754

[29] Cooper LA , Gutman DA , Chisolm C , et al. The tumor microenvironment strongly impacts master transcriptional regulators and gene expression class of glioblastoma. Am J Pathol. 2012;180:2108‐2119. 22440258 10.1016/j.ajpath.2012.01.040 PMC3354586

[30] Şenbabaoğlu Y , Gejman RS , Winer AG , et al. Tumor immune microenvironment characterization in clear cell renal cell carcinoma identifies prognostic and immunotherapeutically relevant messenger RNA signatures. Genome Biol. 2016;17:231. 27855702 10.1186/s13059-016-1092-z PMC5114739

[31] Pereira SS , Monteiro MP , Bourdeau I , Lacroix A , Pignatelli D . Mechanisms of endocrinology: cell cycle regulation in adrenocortical carcinoma. Eur J Endocrinol. 2018;179:R95‐R110. 29773584 10.1530/EJE-17-0976

[32] Subramanian C , Cohen MS . Over expression of DNA damage and cell cycle dependent proteins are associated with poor survival in patients with adrenocortical carcinoma. Surgery. 2019;165:202‐210. 30413320 10.1016/j.surg.2018.04.080 PMC7226676

[33] Yan ZC , He L , Qiu JH , et al. LncRNA HOTAIR participates in the development and progression of adrenocortical carcinoma via regulating cell cycle. Eur Rev Med Pharmacol Sci. 2018;22:6640‐6649. 30402836 10.26355/eurrev_201810_16139

[34] Robbins HL , Hague A . The PI3K/Akt pathway in tumors of endocrine tissues. Front Endocrinol. 2016;6:188. 10.3389/fendo.2015.00188 PMC4707207 26793165

[35] Lee M , Waser B , Reubi JC , Pellegata NS . Secretin receptor promotes the proliferation of endocrine tumor cells via the PI3K/AKT pathway. Mol Endocrinol. 2012;26:1394‐1405. 22692904 10.1210/me.2012-1055 PMC5416982

[36] Fiorentini C , Grisanti S , Cosentini D , et al. Molecular drivers of potential immunotherapy failure in adrenocortical carcinoma. J Oncol. 2019;2019:1‐7. 10.1155/2019/6072863 PMC6463568 31057613

[37] Jasim S , Habra MA . Management of adrenocortical carcinoma. Curr Oncol Rep. 2019;21:20. 30798468 10.1007/s11912-019-0773-7

[38] Gaddy‐Kurten D , Tsuchida K , Vale W . Activins and the receptor serine kinase superfamily. Recent Prog Horm Res. 1995;50:109‐129. 7740154 10.1016/b978-0-12-571150-0.50010-x

[39] Chen W , Zhang J . Potential molecular characteristics in situ in response to repetitive UVB irradiation. Diagn Pathol. 2016;11:129. 27829444 10.1186/s13000-016-0579-y PMC5103495

[40] Sarhan D , Wang J , Sunil Arvindam U , et al. Mesenchymal stromal cells shape the MDS microenvironment by inducing suppressive monocytes that dampen NK cell function. JCI Insight. 2020;5:e130155. 10.1172/jci.insight.130155 PMC7141401 32045384

[41] Nafia I , Toulmonde M , Bortolotto D , et al. IDO targeting in sarcoma: biological and clinical implications. Front Immunol. 2020;11:274. 32194552 10.3389/fimmu.2020.00274 PMC7066301

[42] Geiman TM , Durum SK , Muegge K . Characterization of gene expression, genomic structure, and chromosomal localization of Hells (Lsh). Genomics. 1998;54:477‐483. 9878251 10.1006/geno.1998.5557

[43] Thijssen PE , Ito Y , Grillo G , et al. Mutations in CDCA7 and HELLS cause immunodeficiency–centromeric instability–facial anomalies syndrome. Nat Commun. 2015;6:7870. 26216346 10.1038/ncomms8870 PMC4519989

[44] Geiman TM , Muegge K . Lsh, an SNF2/helicase family member, is required for proliferation of mature T lymphocytes. Proc Natl Acad Sci USA. 2000;97:4772‐4777. 10781083 10.1073/pnas.97.9.4772 PMC18308

[45] Shin SC , Lee KM , Kang YM , et al. Differential expression of immune‐associated cancer regulatory genes in low‐ versus high‐dose‐rate irradiated AKR/J mice. Genomics. 2011;97:358‐363. 21266193 10.1016/j.ygeno.2011.01.005

[46] Yang Q , Tang J , Pei R , et al. Host HDAC4 regulates the antiviral response by inhibiting the phosphorylation of IRF3. J Mol Cell Biol. 2019;11:158‐169. 29800227 10.1093/jmcb/mjy035 PMC6734143

[47] Moheimani F , Koops J , Williams T , et al. Influenza A virus infection dysregulates the expression of microRNA‐22 and its targets; CD147 and HDAC4, in epithelium of asthmatics. Respir Res. 2018;19:145. 30068332 10.1186/s12931-018-0851-7 PMC6090696

[48] Shao L , Hou C . miR‐138 activates NF‐κB signaling and PGRN to promote rheumatoid arthritis via regulating HDAC4. Biochem Biophys Res Commun. 2019;519:166‐171. 31492495 10.1016/j.bbrc.2019.08.092

[49] Xu N , Ke ZB , Lin XD , et al. Identification of survival‐associated alternative splicing events and signatures in adrenocortical carcinoma based on TCGA SpliceSeq data. Aging. 2020;12:4996‐5009. 32217810 10.18632/aging.102924 PMC7138552

